# Metatranscriptome analysis reveals host-microbiome interactions in traps of carnivorous Genlisea species

**DOI:** 10.3389/fmicb.2015.00526

**Published:** 2015-07-14

**Authors:** Hieu X. Cao, Thomas Schmutzer, Uwe Scholz, Ales Pecinka, Ingo Schubert, Giang T. H. Vu

**Affiliations:** ^1^Department of Breeding Research, Leibniz Institute of Plant Genetics and Crop Plant Research (IPK)Gatersleben, Germany; ^2^Department of Plant Breeding and Genetics, Max Planck Institute for Plant Breeding Research (MPIPZ)Köln, Germany; ^3^Faculty of Science and Central European Institute of Technology, Masaryk UniversityBrno, Czech Republic

**Keywords:** Genlisea, plant carnivory, lobster pot trapping, metatranscriptomics, RNA-sequencing, whole-genome gene transcription analysis, algae commensalism, plant-microbe interaction

## Abstract

In the carnivorous plant genus *Genlisea* a unique lobster pot trapping mechanism supplements nutrition in nutrient-poor habitats. A wide spectrum of microbes frequently occurs in *Genlisea's* leaf-derived traps without clear relevance for *Genlisea* carnivory. We sequenced the metatranscriptomes of subterrestrial traps vs. the aerial chlorophyll-containing leaves of *G. nigrocaulis* and of *G. hispidula*. Ribosomal RNA assignment revealed soil-borne microbial diversity in *Genlisea* traps, with 92 genera of 19 phyla present in more than one sample. Microbes from 16 of these phyla including proteobacteria, green algae, amoebozoa, fungi, ciliates and metazoans, contributed additionally short-lived mRNA to the metatranscriptome. Furthermore, transcripts of 438 members of hydrolases (e.g., proteases, phosphatases, lipases), mainly resembling those of metazoans, ciliates and green algae, were found. Compared to aerial leaves, Genlisea traps displayed a transcriptional up-regulation of endogenous NADH oxidases generating reactive oxygen species as well as of acid phosphatases for prey digestion. A leaf-vs.-trap transcriptome comparison reflects that carnivory provides inorganic P- and different forms of N-compounds (ammonium, nitrate, amino acid, oligopeptides) and implies the need to protect trap cells against oxidative stress. The analysis elucidates a complex food web inside the Genlisea traps, and suggests ecological relationships between this plant genus and its entrapped microbiome.

## Introduction

Carnivory, including trapping and subsequent digestion of prey, has evolved several times in plants. About 800 species from five angiosperm orders (Albert et al., 1992; Ellison and Gotelli, 2009) are known to be carnivorous. Although carnivorous plants are distributed worldwide, their occurrence is ecologically restricted to open, wet, nutrient-poor habitats. This indicates that the nutritional benefit from carnivory supports survival of carnivorous plants in such environments. On the other hand, high costs for maintenance of trapping organs and reduced photosynthetic capacity exclude botanical carnivores from most other habitats (Soltis et al., 1999; Farnsworth and Ellison, 2008; Fedoroff, 2012; Król et al, 2012).

*Lentibulariaceae*, the largest monophyletic carnivorous plant family, comprises three genera, *Pinguicula, Utricularia* and *Genlisea*, with three different trapping mechanisms (Jobson et al., 2003; Muller et al., 2006). Similarly to Drosera, the primitive butterwort (Pinguicula) secretes mucilagous adhesive substances in order to capture insects on its leaves (Legendre, 2000). However, Utricularia (bladderwort) and Genlisea (corkscrew plant) use modified leaves either as suction traps (Utricularia) or as lobster pot traps (Genlisea). The bladder-like suction traps of Utricularia generate a water flow that carries small prey (e.g., Daphnia species) within 10 −15 ms into the bladder (Vincent et al., 2011). The prey is digested inside the bladder by means of numerous hydrolases and reactive oxygen species. RNA-seq analysis revealed similar transcriptomes between *Utricularia* vegetative leaves and chlorophyll-free traps (Ibarra-Laclette et al., 2011), but traps contained more transcripts for hydrolytic enzymes for prey digestion and displayed an overexpression of genes involved in respiration compared to aerial photosynthesizing leaves. Colonizing oligotrophic white sands and moist outcrops in tropical Africa and South America, rootless Genlisea species evolved corkscrew shaped subterranean traps to catch protozoa and small metazoa (Barthlott et al., 1998; Plachno et al., 2007; Fleischmann et al., 2010). Trap inward-pointing hairs prevent prey escape and allow only one-way movement toward the “digestion chamber”. Numerous secretory glands in traps apparently produce hydrolases such as acid phosphatases, proteases and esterases in order to digest prey to gain additional N, P and minerals (Adamec, 1997; Ellison and Gotelli, 2001). In spite of detailed knowledge of Genlisea trap anatomy, the complexity of interactions within lobster traps is still not well understood, for instance whether the prey needs to be actively motile to invade traps or whether a passive invasion via a liquid turn-over is also possible. There are multiple reports on specialized organisms surviving and propagating in the traps of carnivorous plants (Siragusa et al., 2007; Peterson et al., 2008; Adlassnig et al., 2011; Koopman and Carstens, 2011; Krieger and Kourtev, 2012). Inside the *Utricularia* and *Genlisea* traps, diverse microbial communities, mainly comprising bacteria, algae, protozoa and rotifers, could live as epiphytes or parasites or might support plant fitness in the context of prey digestion before or without becoming digested themselves (Skutch, 1928; Jobson and Morris, 2001; Richards, 2001; Sirová et al., 2003, 2009; Płachno et al., 2005; Adamec, 2007; Plachno and Wolowski, 2008; Caravieri et al., 2014). So far, little is known about host-microbiome interactions other than microbe's role as source of nutrients, and about possible mutually beneficial impacts of entrapped microbes and their host species. Nevertheless, soil microbes which are associated with root systems of plants (named as root or rhizosphere microbiomes) or live inside plants (named as bacterial/microbial endophytes) have been shown to be important for plant growth and health (for review see Lugtenberg and Kamilova, 2009; Reinhold-Hurek and Hurek, 2011; Berendsen et al., 2012; Rout and Callaway, 2012; Bakker et al., 2013; Vandenkoornhuyse et al., 2015). On the other hand, increasing evidence from different plant systems suggest that plants predominantly influence and modulate the root microbial communities by the active secretion of compounds in so-called root exudates (Broeckling et al., 2008; Badri et al., 2013; Kierul et al., 2015). Moreover, specialized soil microbes with high biomass-degrading capacity could be selected or cultivated, for example in an herbivore microbiome of the leaf-cutter ant (*Atta colombica*) (Suen et al., 2010).

**Figure 1:**
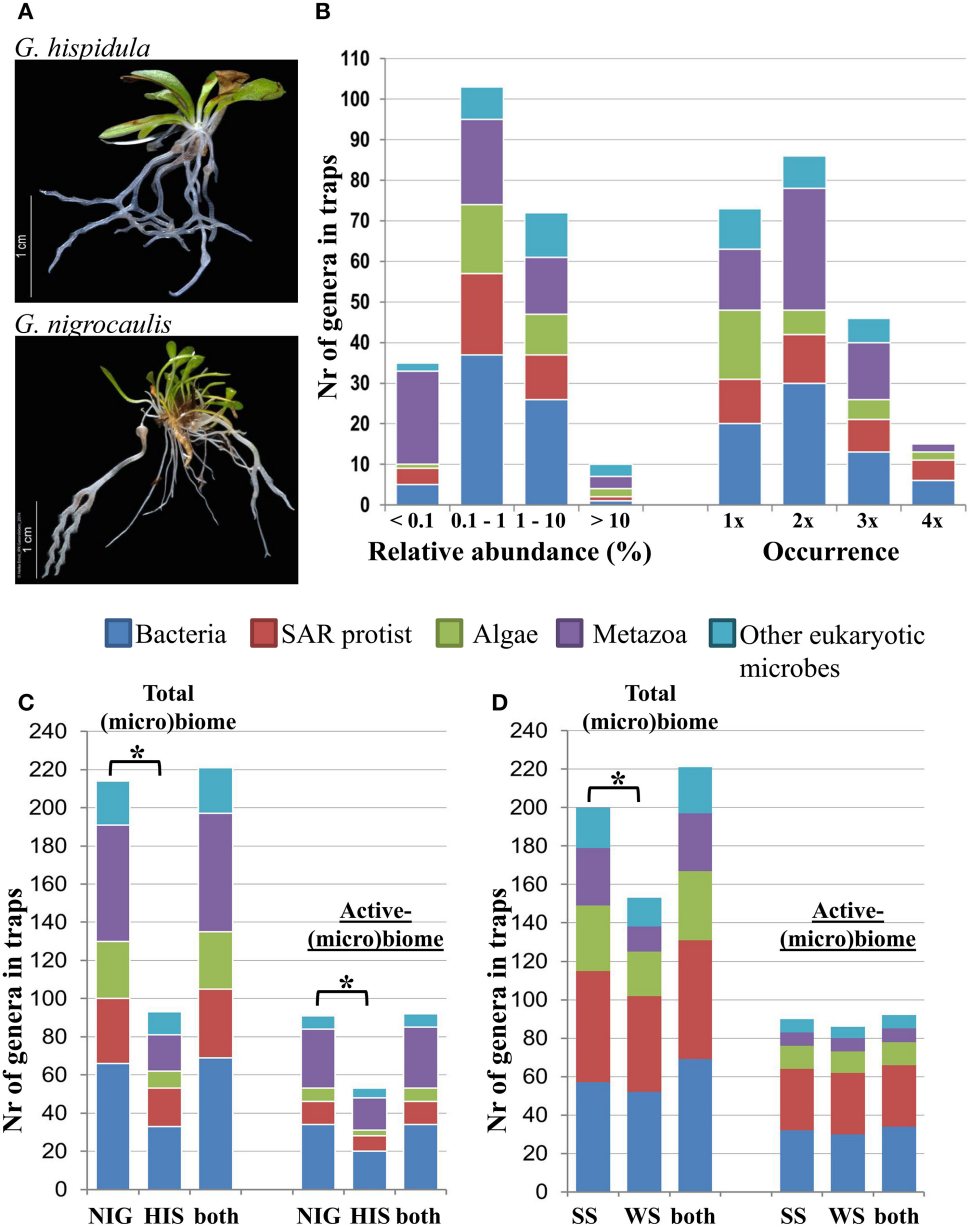
**Morphology and (micro)biome composition in Genlisea traps. (A)** *G. hispidula* has only filiform rhizophylls, while *G. nigrocaulis* displays a trap dimorphism with thick, short-stalked surface traps and filiform, long-stalked deep-soil traps. **(B)** Relative abundance and occurrence of microbe genera of five categories: bacteria, SAR protists (Stramenopiles, Alveolata, and Rhizaria), metazoans and other eukaryotic microbes. Occurrence reflects the number of times a specific genus is found across the 8 different Genlisea metatranscriptome libraries. **(C,D)** Number of genera in Genlisea traps according to species **(C)** or season **(D)**. The active-(micro)biome of Genlisea traps containing preferentially entrapped genera is defined as (i) ≥0.1% relative abundance among each of the five categories; (ii) occurred at least in two trap samples regardless of species or seasonal sampling time; and (iii) trap enrichment with ≥2-fold-change of abundance between traps and leaves. Asterisk indicates significant difference (*p* < 0.05, paired Student's *t*-Test). HIS, *G. hispidula*; NIG, *G. nigrocaulis*; SS, summer season; WS, winter season.

A trap dimorphism has been described for several *Genlisea* species (Studnicka, 1996; Fleischmann, 2012), e.g., for *G. nigrocaulis*, which possesses thick, short-stalked surface traps and filiform, long-stalked deep-soil traps (Figure 1A). In contrast, *G. hispidula* traps are all filiform. Whether different traps contain specific soil microbial communities is still an open question. Here we present, based on a metatranscriptomics approach, a comprehensive diversity characterization of microbial food webs inside the *G. nigrocaulis* and *G. hispidula* traps under homogeneous laboratory conditions. Ribosomal RNA reads, ribotags, from deep sequencing libraries were used to define an “active” community composition across kingdoms which was not achieved in previous studies on prey composition in Genlisea species. In order to investigate profound plant-microbe interactions in the Genlisea trap environment, active metabolic pathways of the entrapped microbiome were reconstructed and Genlisea trap-specific and differentially expressed transcripts were analyzed.

## Materials and methods

### Plant sampling, RNA isolation and sequencing

*G. nigrocaulis* STEYERM and *G. hispidula* STAPF [obtained from commercial sources: Best Carnivorous Plants (bestcarnivorousplants.com), Merzig (carnivorsandmore.de) and Nüdlingen (falle.de)] were cultivated in the greenhouse of the IPK Gatersleben, Germany. Plants were grown in pots with a mixture of peat and sand. The soil was kept wet by rain water, containing small organisms living naturally inside. Leaves and traps of both species were collected in summer season 2010 (SS) and winter season 2011 (WS) after thorough cleaning with 2 l of running cold distilled water. Total RNA samples were isolated using RNeasy Kit (Qiagen) with DNaseI treatment. The sample quality was controlled on a 2100 Bioanalyzer (Agilent). Illumina RNA-TruSeq libraries were prepared from 1 μg RNA of each sample without mRNA enrichment or rRNA depletion. Illumina Hiseq2000 paired-end sequencing (2x100 bp reads, 200 bp insert size) resulted in at least 31 million reads per library (Table 1). The raw RNA-seq data is deposited in the project “PRJEB1867” at the European Nucleotide Archive (www.ebi.ac.uk/ena/).

**Table 1:** **Summary of RNA-sequencing output and read mapping analysis**.

**Sample name**	**Species**	**Season**	**Organ**	**SRA IDs**		**Total high quality reads**	**Library proportion**					
							**rRNA reads^a^**		**Genlisea reads^b^**		**Non-host mRNA reads^c^**	
				**Experiment**	**Sample**		**Read number**	**% of reads**	**Read number**	**% of reads**	**Read number**	**% of reads**
NIG_SS_t	*G. nigrocaulis*	Summer	Trap	ERX272583	ERS257172	55,570,966	122,734 (218,975)	0.22 (0.39)	18,190,523	32.73	6,790,717	12.22
NIG_SS_l	*G. nigrocaulis*	Summer	Leaf	ERX272581	ERS257171	68,905,010	290,521 (492,631)	0.42 (0.71)	39,416,889	57.20	148,530	0.22
NIG_WS_t	*G. nigrocaulis*	Winter	Trap	ERX272584	ERS257172	39,914,128	195,871 (297,182)	0.49 (0.74)	17,107,634	42.86	2,811,952	7.05
NIG_WS_l	*G. nigrocaulis*	Winter	Leaf	ERX272582	ERS257171	31,504,414	127,178 (194,034)	0.40 (0.62)	21,144,415	67.12	10,564	0.03
HIS_SS_t	*G. hispidula*	Summer	Trap	ERX272589	ERS257479	83,721,886	131,033 (203,470)	0.16 (0.24)	13,312,311	15.90	40,547	0.05
HIS_SS_l	*G. hispidula*	Summer	Leaf	ERX272587	ERS257478	73,706,164	41,958 (72,126)	0.06 (0.1)	12,170,713	16.51	17,254	0.02
HIS_WS_t	*G. hispidula*	Winter	Trap	ERX272590	ERS257479	60,971,788	91,577 (133,429)	0.15 (0.22)	8,733,316	14.32	2,783	0.005
HIS_WS_l	*G. hispidula*	Winter	Leaf	ERX272588	ERS257478	60,849,726	80,315 (124,529)	0.13 (0.20)	7,471,549	12.28	538,121	0.88

a *SILVA LSURef_115 and SSURef_NR99_115 sequences were used as reference for read mapping with minimal 97% similarity (or minimal 80% similarity in brackets)*.

b *Annotated G. nigrocaulis genome sequences were used as reference for read mapping with minimal 80% similarity (the G. nigrocaulis genome is 18 times smaller and has one third of the gene number compared to G. hispidula)*.

c *Non-redudant and trap-specific de novo assembled contigs (≥1 kbp) of G. nigrocaulis trap libraries after filtering out rRNA or Genlisea gene containing contigs were used as reference for read mapping with at least 80% similarity*.

### Taxonomic assignment of RNA-seq reads

RNA-Seq reads from total RNA libraries were trimmed for sequence quality using the standard pipeline (quality limit 0.05, minimum read length 80) of the CLC Genomics Workbench v5.5.1 (CLC bio, Cambridge, MD). Using the RNA-seq module of the CLC Genomics Workbench, trimmed and high quality reads from each dataset were mapped to the non-redundant and truncated version of the ribosomal RNA SILVA reference sequences [LSURef_115 and SSURef_NR99_115, (Quast et al., 2013)]. With standard mapping parameters (minimum length 90% and minimum similarity 80%), on average 0.4% reads of each library could be mapped to rRNA reference sequences (Table 1). In order to remove potentially false assignment, more strict mapping parameters with minimum similarity 97% were applied. Mapping outputs (total mapped reads) of SILVA reference sequences which were mapped by at least one unique read were summarized for each phylotype using the SILVA taxonomy description by MEGAN software (v 5.8.6, Huson et al., 2011). Taxonomy rarefaction plot was performed in MEGAN for all bacterial taxa (Figure S1). For taxonomic affiliation, ribosomal sequences of eukaryotic cellular organelles (mitochondria, chloroplast) were not taken into account.

Relative abundance (read count per total million reads) and reoccurrence of each assigned genus were categorized as Bacteria, SAR protozoans (Stramenopiles, Alveolata, and Rhizaria), green algae (Chlorophyta), metazoan or other eukaryote groups. For each category, a relative abundance cutoff of 0.1% and at least appearance within two samples was applied at genus level for each library. Trap enrichment was calculated as the fold change in abundance of each phylotype between trap sample and its corresponding leaf sample. For every phylotype, a paired *t*-test was used to determine significant differences for pairwise comparisons between trap and leaf samples of each plant species, and for the winter season vs. the summer season (seasonal effect). NCBI Taxonomy IDs of assigned genera were extracted by the Tax Identifier tool (http://www.ncbi.nlm.nih.gov/Taxonomy/TaxIdentifier/tax_identifier.cgi) and used for drawing a phylogenetic tree by the phyloT tree generator (http://phylot.biobyte.de) and iTOL graphical editor (http://itol.embl.de/).

### Clustering and phenotype enrichment analysis in comparison with reference environmental datasets

The same taxonomy assignment pipeline was applied for 18 published metatranscriptome Illumina sequencing datasets of creek, soil, feces, marine sediment, marine water body and lake habitats (Table S1, Caporaso et al., 2011). A total of 13,246 bacterial SILVA reference sequences have at least one unique mapped read in one dataset. UPMA clustering analysis of bacteria diversity in all datasets with the Bray-Curtis matrix was performed with all bacterial taxa by using MEGAN software (v 5.8.6). Bacterial phylotypes with corresponding read counts were imported into METAGENassist (Arndt et al., 2012, www.metagenassist.ca) for mapping bacterial phenotypic information. Several phenotype categories including oxygen requirement, energy source, metabolism and habitat may have multiple phenotypic traits associated with a given taxon. A paired *t*-test was used to examine differences in species richness and intra-group similarity between different attributes such as organs, species and seasons.

### *De novo* assembly and analysis of trap-specific community transcriptomes

Trimmed and high quality reads from each *G. nigrocaulis* library were separately *de novo* assembled by the CLC Genomics Workbench 5.5.1 with automatic bubble and word sizes and minimal 200 bp contig length. Contigs longer than 500 bp were sequentially filtered out of *G. nigrocaulis* high and low confidence transcripts (Vu et al., unpublished), SILVA LSURef_115 and SSURef_NR99_115 sequences by using ublast (1E-09) of the Usearch software (v 7.0.1090_win32, (Edgar, 2010). The remaining contigs from trap samples were clustered at 80% identity by Usearch and subsequently filtered out from (ublast, 1E-09) *de novo* assembled contigs of leaf samples, resulting in 31,710 non-redundant microbe transcript contigs longer than 1000 bp.

The 31,710 microbe transcript contigs (in total 51.2 Mbp) served as a reference for read mapping using the RNA-seq module of the CLC Genomics Workbench v5.5.1 with standard mapping parameters (minimum length 0.9 and minimum similarity 0.8) for all 8 Genlisea mRNA-seq datasets (Table 1). Relative abundance (read count per total million reads) and fold change of abundance between trap and leaf were calculated for every contig. A similar analysis was performed using the annotated *G. nigrocaulis* genome (Vu et al., unpublished) as reference. Transcript amounts (in reads per kilobase of exon per million reads) were calculated for every gene and quantile-normalized. Log2 ratios were used to measure relative changes in expression level between each pair of trap and its corresponding leaf sample. Genes were considered expressed if they have (1) more than one unique mapped read and (2) have more than five total mapped reads. Absolute values of the corresponding log2 ratios higher than 2 and the *p*-value of a paired *t*-test (trap vs. leaf) lower than 0.05 are conditions for selecting differentially expressed genes.

### Functional annotation of differentially transcribed genes and enrichment analysis

By using Blast2GO (Conesa and Gotz, 2008), 12,564 microbe transcripts of 1500–8000 bp length (comprising 27.9 Mbp and corresponding 54.5% of the transcribed microbial sequences) were blasted against the NCBI protein reference sequence (*E*-value cut off 10^−3^) and further annotated with default filtering parameters (*E*-value cutoff 10^−6^, Annotation cutoff 55, GO Weight 5). Generic GO-slim categories were used to provide a summary of GO annotation results. Enzyme code class assignment was exploited to define the list of hydrolases. Species information and bit score of blastx from the best blast hit result of every transcript were exported and taxonomically summarized by LCA algorithm from MEGAN software with a minimum score 50. Phyla which have been detected by ribosomal RNA assignment were used as main categories. Best hits from Chordata species were referred to as the Metazoa group. Enrichment analysis using the Fishers's Exact Test with Multiple Testing Correction of standard false discovery rate (FDR) was carried out in Blast2Go for enriched GO categories with a *p*-value cutoff of 0.05.

## Results and discussion

The Genlisea traps primarily serve as the root-substitutes, anchoring the plant in the soil and absorbing soil-borne nutrients. Importantly, these chlorophyll-free, subterranean rhizophylls are tubular, modified leaves which resemble a lobster pot, retaining numerous and highly diverse microbes and small animals as prey in order to provide complement nutrients *via* carnivorous diet. To identify active players in this semi-closed food web, we examined total RNA from leaves and traps of perennial *G. nigrocaulis* and *G. hispidula* and characterized the trap microbiome by metaRNA sequencing. Extensive washing of the samples prior to RNA extraction was applied in order to remove loosely associated microbes on surfaces of plant tissues. Two winter and summer season replicates of each sample were analyzed.

### Trap-specific enrichment among the highly diverse and dynamic phylotypes of Genlisea traps

Deep sequencing has been shown to be a suitable approach for large-scale comparisons of microbial communities (Caporaso et al., 2011; Yarza et al., 2014). With whole-community RNA sequencing, amplification bias and primer design limitations in rRNA amplicon sequencing approaches can be compensated. Moreover, because of the short mRNA half-life, metatranscriptomics presents abundance information on active populations in the community. By using a stringent mapping approach, we assigned on average 135,148 ribosomal RNA reads of each RNA-seq library to ribosomal RNA SILVA reference sequences with 39–188 phylotypes at genus level (Table 1, Figure 1). On average, microbial communities in *G. nigrocaulis* traps (144–188 genera) were more diverse than in *G. hispidula* (39–73 genera) traps, regardless of seasonal sampling. Overall we found in Genlisea trap samples 184 out of total 220 uniquely detected genera having at least 0.1% relative abundance of either bacteria, SAR protists (Stramenopiles, Alveolata and Rhizaria), green algae (Chlorophyta), metazoa, or other eukaryotic microbes (Figure 1B). The majority of genera (103 out of 184 = 55.9%) in Genlisea traps were rare (0.1–1% abundance), suggesting high sensitivity of the RNA-seq sequencing approach. The dominant genera with >10% of each phylogenetic group include the widespread aerobic soil bacterium *Pedosphaera*, the freshwater ciliate *Tetrahymena*, two freshwater planktonic green algae *Chlamydomonas* and *Carteria*, the minute worm *Aeolosoma, the* predatory flatworm *Stenostomum*, the cosmopolitan oribatid mite *Trhypochthonius*, the aquatic fungus *Entophlyctis*, and two amoebae (the flagellate *Phalansterium* and the lancet-shaped *Paradermamoeba)*. Of 220 detected genera, 33.2% were found only in a single trap sample and only 6.8% were in all trap samples regardless of season and Genlisea species tested. The green algae *Carteria* and the fungus *Entophlyctis* were prevalent in only one sample, while eight other dominant genera appeared in more than one trap sample.

**Figure 2:**
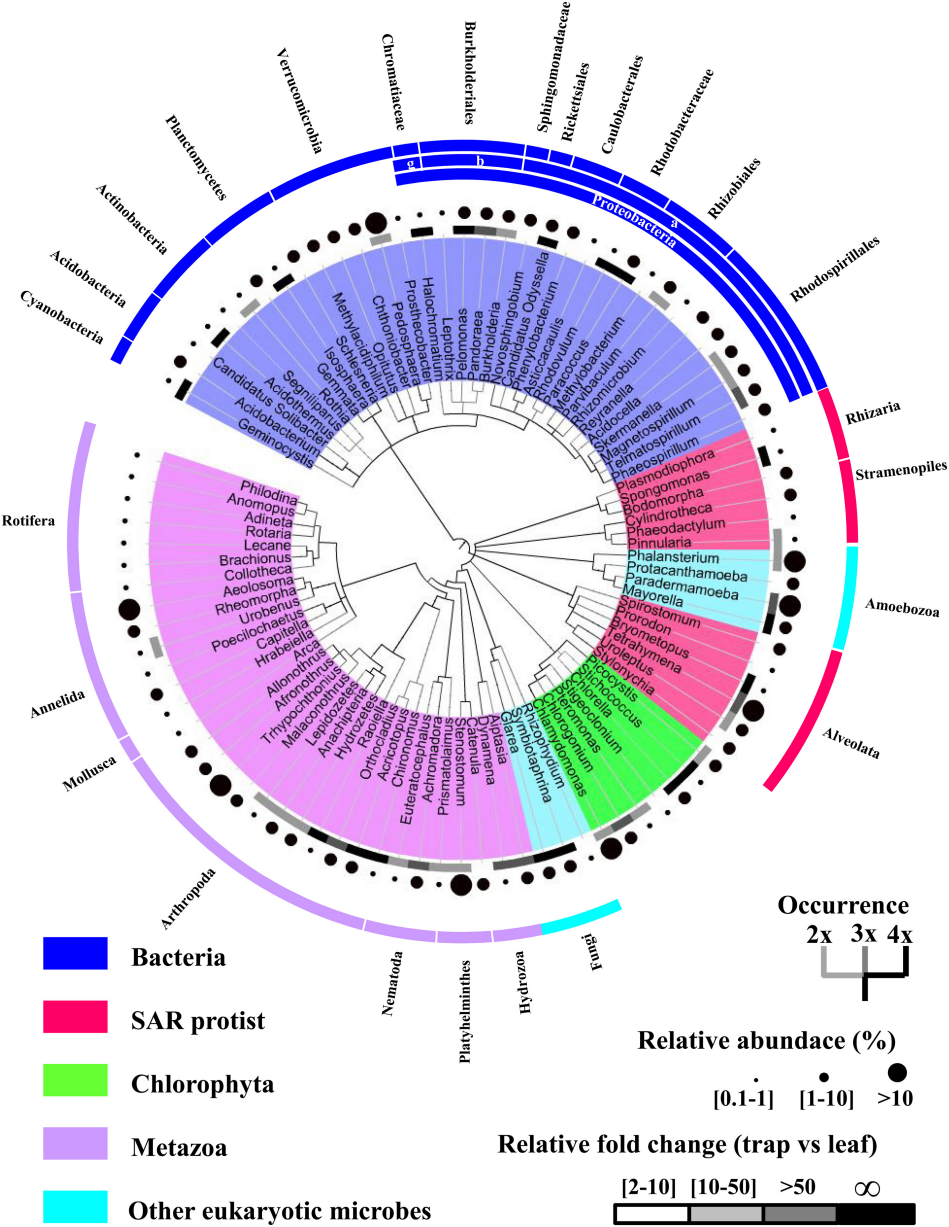
**The active-(micro)biome of Genlisea traps contains 92 preferentially entrapped genera**. Relative abundance, frequency of appearance in samples and relative fold change of abundance in trap vs. leaf are shown. Definition of preferentially trapped genera can be found in the legend of Figure 1. ∞ indicates trap exclusive presence.

Among the 133 genera having 0.1% or higher relative abundance and being found in at least two trap samples, 92 genera belonging to 19 phyla were enriched (two-fold or higher relative abundance) in traps in comparison with corresponding leaves (Figure 2). These preferentially entrapped or trap-enriched organisms, here defined as the active-(micro)biome of Genlisea traps, consist of 34 bacteria, 12 SAR protists, 7 green algae, 32 metazoa, and 7 other eukaryotic genera. Proteobacteria, Chlorophyta (green algae) and Arthropoda represent the most diverse phyla in this community, largely extending the view of Barthlott et al. (1998). These authors proposed that Genlisea species are specialized in capturing protozoans, based on their laboratory experiments and field observations. Microscopic studies on trap content of different cultivated and field collected Genlisea species showed that mites (Acari), roundworms (Nematoda), flatworms (Platyhelminthes), annelids (Annelida) and rotifers (Rotifera) are common prey (Płachno et al., 2005; Fleischmann, 2012). In addition, unicellular algae were also frequently encountered inside of the Genlisea rhizophylls as prey and/or as commensals (Płachno et al., 2005; Plachno and Wolowski, 2008). Our data suggest an even richer bacteria community than the 10 bacterial genera including *Phenylobacterium* and *Magnetospirillum* that were found in 16S rDNA amplification libraries of *Genlisea filiformis* traps collected from natural habitats (Caravieri et al., 2014). Limitation in primer design and amplification bias could result in an underestimation of sequence diversity of 16S rDNA amplification libraries.

Our comparative data indicate that the prey spectrum of the uniform *G. hispidula* traps is less diverse than that of the dimorphic *G. nigrocaulis* traps, although under our cultivation conditions the microfauna composition was likely homogeneous (Figures 1C,D). In *G. nigrocaulis* traps, we detected 31 out of the 32 preferentially entrapped metazoans, except for the polychaete worm Capitella. Interestingly, this worm was repeatedly abundant in the filiform traps of *G. hispidula*, although only 17 out of the 32 metazoan genera occurred there. This corroborates the hypothesis that different Genlisea species may prefer different prey (Studnicka, 1996) or are of different attractivity for potential prey species. Nevertheless, both types of Genlisea traps captured prey of different phyla which are abundant in soil.

To test the effectiveness of our stringent mapping approach, the bacterial composition of Genlisea samples was further analyzed in comparison with published metatranscriptome datasets for various environments including soil, creek, lake, feces, marine water body and marine sediments (Table S1, Figure S1A). As expected, clustering analysis based on abundance of all bacterial taxa indicates that Genlisea samples are more similar to creek, soil, and lake samples than marine sediment, marine water or feces samples (Figure S1B). The relationship between environmental samples using our taxonomy assignment comes in line with the output from the QIIME pipeline (Caporaso et al., 2011). Notably, variation in taxonomic structures between Genlisea samples is higher than other environmental sample groups, except for marine water samples (Figure S1B). In spite of this remarkably dynamic composition, Genlisea traps from same species are more similar to each other and differentiation between Genlisea samples across sampling season is not evident from the cluster dendrogram.

Given that plant root microbiomes vary by soil type and plant species (Haichar et al., 2008; Bulgarelli et al., 2012; Turner et al., 2013; Ofek-Lalzar et al., 2014; Cardinale et al., 2015), a direct comparison with root microbiota and/or rhizosphere of other terrestrial plants might not be meaningful. Nevertheless, following interesting findings are noteworthy in Genlisea-associated bacteria. (i) Similar to microbiota in Arabidopsis' root (Bulgarelli et al., 2012), other plant rhizospheres or bulk soil (Turner et al., 2013), we identified Proteobacteria as the dominant bacterial phylum (from 54.9 to 64.2% bacterial reads) in Genlisea samples (Figure S2). However, Rhodospirillaceae represent the majority (35.9% bacterial reads, 55.9% Proteobacteria reads) in *G. nigrocaulis* traps, whereas these bacteria are largely underrepresented in *G. hispidula* traps and Genlisea leave samples (from 0.8 to 6.9% bacterial reads). Within this purple non-sulfur bacterial family, the chemoheterotrophs include the facultative anaerobic genera Skermanella, Telmatospirillum and the strictly aerobic and microoxic genera Magnetospirillum are mainly found in Genlisea samples. (ii) In Proteobacteria phylum, the acetic acid bacterium Asaia and several genera in plant growth-promoting Rhizobiales are highly enriched in *G. hispidula* traps and Genlisea leave samples. The abundant Asaia genus (from 6.4 to 17.8% bacterial reads) has recently recognized as bacterial symbionts of various insects (Crotti et al., 2009). (iii) Surprisingly, Planctomycetes and Verrucomicrobia, which contain few cultured representatives and are poorly understood, are highly abundant in Genlisea traps but are mostly depleted (compared to bulk soil and rhizosphere) in root-associated bacteria of Arabidopsis and rice (Lundberg et al., 2012; Edwards et al., 2015). Verrumimicrobia are more abundant than Planctomycetes in *G. nigrocaulis* traps (24.9 and 4.2% bacterial reads, respectively). The opposite is found in *G. hispidula* traps with 7.2 and 18.7% bacterial reads, respectively (Figure S2). (iv) A depletion in abundance of Acidobacteria and Firmicutes in Genlisea traps, as compared to Genlisea leaves, suggests preferences of protozoa predators in the trap. However, belonging to Acidobacteria phyla, Acidobacterium and *Candidatus* Solibacter in Genlisea trap's active-microbiome apparently use complex carbon sources and are well equipped to tolerate low-nutrient conditions and fluctuations in soil hydration (Ward et al., 2009).

**Figure 3:**
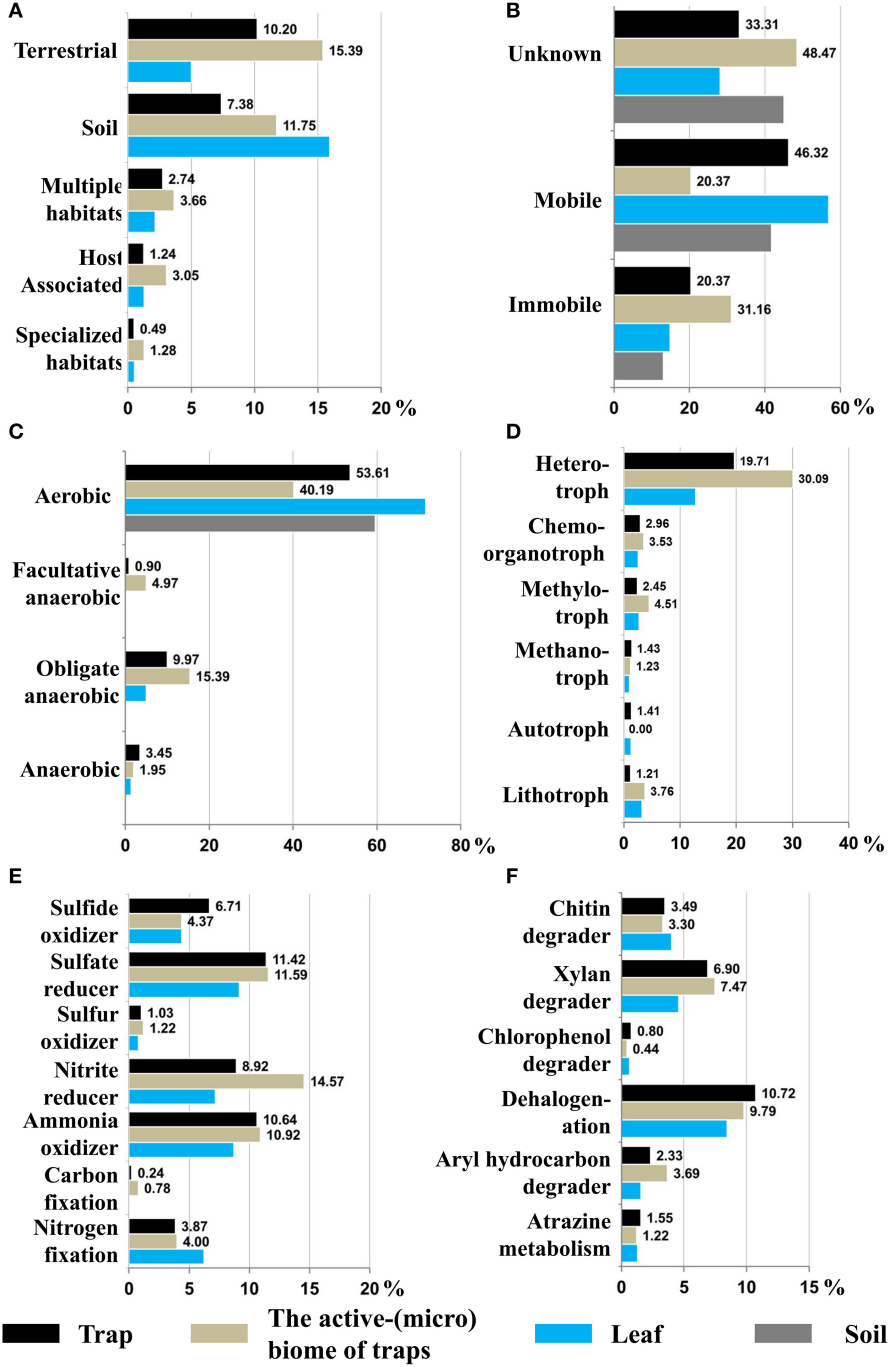
**Phenotype profiling of bacterial communities between Genlisea trap samples vs. Genlisea leaves or soil samples**. Phenotype information of habitat **(A)**, mobility **(B)**, oxygen requirement **(C)**, energy resources **(D)**, and metabolisms **(E,F)** was extracted from the METAGENassist database.

To provide an additional level of functional understanding of the bacterial active-microbiome of Genlisea traps (trap-enriched set), available phenotype information of identified genera from the METAGENassist database (Arndt et al., 2012) was employed. This data suggest that free-living bacteria from terrestrial (10.2%) and soil (7.4%) habitats are dominant in Genlisea traps, while so-called host associated bacteria comprised only 1.2% of trap residents (Figure 3A). Interestingly, among the bacterial active-microbiome of Genlisea traps, the proportions of host-associated and habitat-specific bacteria were increased to 3 and 1.3%, respectively. Of the entrapped bacteria 46.3% were motile and 20.4% were non-motile; among the preferentially trap-enriched bacteria 31.2% were not motile (Figure 3B). So far, several contradictory hypotheses have been published regarding active (Meyers-Rice, 1994; Studnicka, 2003a,c) or passive trapping (Barthlott et al., 1998; Adamec, 2003; Płachno et al., 2005; Plachno and Wolowski, 2008) in Genlisea. The presence of immobile and free-living microbes in Genlisea traps was previously considered as evidence for the hypothesis of an actively drawing bacteria into Genlisea rhizophylls systems (Studnicka, 2003a). Virtually no measurable water flow and lacking bifid glands for water pumping, as occur in Utricularia (Adamec, 2003), rather suggest a passive invasion via a liquid turn-over to explain trapping of immobile bacteria in Genlisea.

Studnicka (2003b) postulated that Genlisea plants attract soil microfauna by transiently creating an oxygen-rich area in their rhizophylls. The presence of bacteria with different oxygen requirements in Genlisea traps (Figure 3C) is in accordance with this hypothesis. Although aerobic bacteria are predominant, facultative and obligate anaerobic bacteria were enriched among the preferentially trapped microbes from 0.9 and 9.97 to 4.97% and 15.39%, respectively. Therefore, bacterial commensals might be adapted to anoxia interrupted by periods of high O_2_. The oxygen concentration was found very small or zero in mature traps of Genlisea by a still unclear mechanism (Adamec, 2007).

Phenotype mapping of energy resources (Figure 3D) revealed that most of trapped bacteria are heterotrophic (19.7%), and that methylotrophic (2.45%) or lithotrophic (1.2%) bacteria were also enriched (Figure 3E). In terms of metabolic activity, Genlisea traps contain small fractions of bacteria with ability for nitrogen (3.87%) or carbon fixation (0.24%). Plant-associated N_2_ fixation has been considered as a potential source of N for carnivorous plants with pitcher or snapping traps (Prankevicius and Cameron, 1991; Albino et al., 2006). Although N_2_ fixing bacteria represent up to 16% of the bacterial community in *Utricularia* traps, N_2_ fixation contributed less than 1% of daily N gain of *Utricularia* (Sirova et al., 2014). This limited N_2_ fixation is likely due to the high concentration of NH_4−_N in the *Utricularia* trap fluid, resulting from fast turnover of organic matter. In *Genlisea* traps, bacterial ammonia oxidizing or nitrite reducing bacteria are abundant with 10.6 and 8.9%, respectively. This suggests a close interaction of nitrifying and denitrifying bacteria in the nitrogen cycling within this microbial community. In rice paddy soils, nitrite oxidizers were abundant in rice roots and its rhizospheric soil, however ammonia oxidizers were dominant in surface soil (Ke et al., 2013). Furthermore, in Genlisea rhizophylls, there are several bacteria groups with various degrading capacity (Figure 3F), including dehalogenation (10.7%), chitin degradation (3.49%), and xylan degradation (6.9%).

### Contribution of microbial mRNA to the Genlisea trap meta-transcriptome

With a glimpse of mechanistic understanding of the trap microbiomes from the METAGENassist database, we further explored the contribution of microbes to Genlisea carnivory by studying mRNA transcripts of traps. The metatranscriptome of each *G. nigrocaulis* mRNA-seq dataset was *de novo* assembled and the contigs containing ribosomal RNA and Genlisea transcripts were filtered out, resulting in a set of 31,710 non-plant transcripts (51.2 Mbp). The main fraction of non-plant transcripts ranging from 1500 to 8000 bp (12,564 contigs, 27.9 Mbp) was analyzed. A total of 10,518 transcripts had significant BLAST hits (*E* ≤ 1.0E-3) in the NCBI protein reference database (Tables S2, S3). Of these, 10,501 transcripts could be taxonomically assigned by the LCA algorithm in MEGAN (minimal blast bit score of 50). The highest percentage of top blast hits came from metazoan species (73.6%) including Arthropoda (20.2%), Mollusca (12.8%), Nematoda (2.6%), probably indicating that Genlisea plants lack of voracious mechanisms to kill trapped large-sized preys. Interestingly, green algae, bacteria, Amoebozoa and Alveolata species contribute to 5.7, 4.3, 3.8, and 2.5% respectively, of transcripts of the Genlisea trap microbe transcriptome. In total, 16 out of 19 phyla, which, according to their rRNA, were preferentially enriched in the traps, apparently contribute to the active mRNA meta-transcriptome.

Of the 10,518 microbe transcripts, 6140 transcripts could be annotated (*E*-value hit filter of 1.0E-6, annotation cutoff of 55), and 1298 transcripts could be further assigned with an enzyme code. The top five KEGG (Kyoto Encyclopedia of Genes and Genomes) pathways of this microbial metatranscriptome include purine metabolism (181 transcripts, 29 enzymes), prokaryotic carbon fixation pathways (57 transcripts, 15 enzymes), pyruvate metabolism (54 transcripts, 17 enzymes), thiamine metabolism (52 transcripts, 1 enzyme as nucleoside-triphosphate phosphatase EC 3.6.1.15) and the tricarboxylic acid (TCA) cycle (48 transcripts, 15 enzymes). In total, only 15 transcripts (1.1% of all EC assigned transcripts) with assigned enzyme codes had a significant best hit from bacterial species, although the proportion of bacterial transcripts is in the same range as those from algae, amoebes, or ciliates. In total, 408 bacterial transcripts having *E*-value less than 1E-3 and a bit score higher than 50, originated from 300 bacterial species of 214 genera, belonging mainly to the most abundant bacteria phyla Proteobacteria (151 transcripts), Cyanobacteria (76 transcripts) and Firmicutes (75 transcripts).

The enzyme code distribution of the microbial transcriptome showed 33.7% transcripts encoding hydrolases (Tables 2, S2). Main contributors of hydrolases were metazoans (80.1%), Alveolata (8%), green algae (5.7%), and Amoebozoa (1.14%). Especially, phosphatases (EC 3.1.3) are hydrolases of interest because prey likely provides supplemental phosphate to carnivorous plants in poor habitats (Adamec, 1997). High extracellular phosphatase activity was detected in glandular structures of Genlisea traps as well as in Chlamydomonas sp. living inside Genlisea traps (Plachno et al., 2006; Plachno and Wolowski, 2008). We found in the microbial metatranscriptome 86 phosphatases mainly from Metazoa (73 contigs), Alveolata (5 contigs), Chlorophyta (3 contigs), and Amoebozoa (2 contigs). These groups also contribute to the pool of peptidases-encoding transcripts (EC 3.4), with 77.8, 12.3, 6.2, and 1.2%, respectively. Dominant or co-dominant species for the three protist groups in terms of mRNA transcript abundance are *Tetrahymena thermophila* (204 transcripts, 76.7% transcripts of Alveolata), *Volvox carteri f. nagariensis* (247 transcripts, 41.2% transcripts of Chlorophyta), *Chlamydomonas reinhardtii* (196 transcripts, 32.7% transcripts of Chlorophyta), *Acanthamoeba castellanii* str. Neff (164 transcripts, 41.6% transcripts of Amoebozoa) and *Dictyostelium purpureum* (110 transcripts, 27.9% transcripts of Amoebozoa). Given the limited availability of genomic data for unicellular Eukarya, it is more likely that transcripts could have been from soil-borne related species. Among the whole microbiome, *T. thermophila* which is a voracious predator of bacteria (Eisen et al., 2006), showed 16 enriched GO terms, including hydrolase activity (FDR 1.0E-12), peptidase activity (FDR 4.7E-4), and pyrophosphatase activity (FDR 1.2E-3) confirmed by the Fisher's Exact Test (Table S4). From two green algae, transcripts required for photosynthesis (FDR 3.2E-6 and 3.8E-3 for *V. carteri* and *C. reinhardtii*, respectively) were accumulated. Enrichment of transcripts involved in transmembrane transport (FDR 2.7E-3) and other substance transport mechanisms (“single-organism transport,” FDR 3.8E-3) were observed in *C. reinhardtii*, while *V. carteri* produced transcripts enriched for generation of precursor metabolites and energy (FDR 2.5E-3) and for stress response (FDR 0.042). No statistically significant enrichment was found comparing transcripts of *Acanthamoeba castellanii* str. Neff, *Dictyostelium purpureum*, or of all bacteria species with the whole microbial transcriptome.

**Table 2:** **List of microbe hydrolases (EC:3.1.3)**.

**Contig ID**	**Trap Abundance^a^**	**Description**	**EC**	**Sequence length**	**Best hit from blast search**		
					**Protein ID**	***E*-value**	**Species name**
microbe_contig_1801	3.93	Alkaline tissue-non-specific isozyme	EC:3.1.3.1	2305	XP_002919566	2.42E-144	Ailuropoda melanoleuca^b^
microbe _contig_9779	3.85	Testicular acid phosphatase	EC:3.1.3.5	2202	NP_001013355	4.79E-25	Danio rerio^b^
microbe _contig_25278	2.44	Fructose- - cytosolic-like	EC:3.1.3.11	1604	XP_001007592	0	Tetrahymena thermophila
microbe _contig_19528	1.62	Fructose- - chloroplastic-like	EC:3.1.3.23	1848	XP_005821930	1.26E-171	Guillardia theta CCMP2712
microbe _contig_69095	0.97	Phosphatidylinositide phosphatase sac1	EC:3.1.3.64; EC:3.1.3	2234	XP_007505127	0	Monodelphis domestica^b^
microbe _contig_47809	0.86	6-phosphofructo-2-kinase fructose- -bisphosphatase isoform x2	EC:3.1.3.46; EC:2.7.1.105	1863	XP_002404032	0	Ixodes scapularis
microbe _contig_36107	0.85	Delta-aminolevulinic acid chloroplastic	EC:4.2.1.24; EC:3.1.3.11	1659	XP_001701779	0	Chlamydomonas reinhardtii
microbe _contig_42194	0.71	Phosphatidylinositol -trisphosphate 3-phosphatase tpte2-like isoform x1	EC:3.1.3.67	2420	XP_005516390	3.04E-129	Pseudopodoces humilis^b^
microbe _contig_30058	0.65	Ser thr phosphatase family protein	EC:3.1.3.2	1613	XP_001027093	0	Tetrahymena thermophila
microbe _contig_45862	0.58	Cytosolic purine 5 -nucleotidase isoform x3	EC:3.1.3.5	2114	XP_008551228	0	Microplitis demolitor
microbe _contig_27837	0.56	6-phosphofructo-2-kinase fructose- -bisphosphatase 1 isoform 2	EC:3.1.3.46; EC:2.7.1.105	1670	XP_001634601	7.44E-66	Nematostella vectensis
microbe _contig_12875	0.51	Enolase-phosphatase e1	EC:3.1.3.77	1797	XP_001493062	2.39E-79	Equus przewalskii^b^
microbe _contig_91384	0.43	Lysosomal acid phosphatase precursor	EC:3.1.3.2	2429	NP_001013355	1.38E-56	Danio rerio^b^
microbe _contig_4435	0.39	Deubiquitinating protein vcip135	EC:3.1.3	2141	XP_006006858	1.58E-165	Latimeria chalumnae^b^
microbe _contig_80314	0.31	Inositol-tetrakisphosphate 1-kinase	EC:2.7.1.159; EC:2.7.1; EC:2.7.1.134; EC:3.1.3	2112	XP_007252574	3.08E-81	Astyanax mexicanus^b^
microbe _contig_42454	0.29	Bifunctional polynucleotide phosphatase kinase-like	EC:3.1.3.32; EC:2.7.1.78	1958	XP_004336369	9.31E-100	Acanthamoeba castellanii str. Neff
microbe _contig_98753	0.27	3 (2) -bisphosphate nucleotidase-like	EC:3.1.3.7; EC:3.1.3.57	1535	XP_001690049	1.25E-125	Chlamydomonas reinhardtii
microbe _contig_105139	0.23	Phosphoglycolate phosphatase	EC:3.1.3.18	1658	XP_003624218	2.93E-104	Medicago truncatula
microbe _contig_1444	0.18	Fructose- - cytosolic-like	EC:3.1.3.11	1581	XP_001007592	0	Tetrahymena thermophila

a *Trap abundance was calculated as quantile-normalized expression values (in read per kilobase of exon per million read units) for G. nigrocaulis trap samples*.

b *Sequences were considered as from metazoan species because of lacking genomic reference sequences*.

### The rhizophyll transcriptome of *G. nigrocaulis*

Using the annotated *G. nigrocaulis* genome as reference, the Genlisea rhizophyll trancriptomes were characterized by RNA sequencing analysis in comparison to the corresponding leaf transcriptome. Samples of *G. nigrocaulis* and *G. hispidula* from two different seasons were included (Table 1). Relative to leaf samples, 1098 transcripts were differentially transcribed (*p* < 0.05) in *G. nigrocaulis*. Hence, 6.4% of all 17,113 *G. nigrocaulis* genes, corresponding to 8.5% of genes transcribed either in leaves or traps, were differentially expressed. Of the 1098 differentially expressed genes (DEGs), 69 showed an at least two-fold accumulation or reduction of transcripts (Table 3). When comparing trap and leaf samples of *G. hispidula* by mapping RNA-seq reads of *G. hispidula* to the genome of *G. nigrocaulis*, in total 306 differentially expressed genes were found, and 33 of these revealed an at least two-fold different abundance. The difference, compared to the situation found in *G. nigrocaulis*, could be explained by divergence of transcript sequences between two species, resulting in a less efficient read mapping (Table 1). The *G. hispidula* genome is allotetraploid and 18 times larger than that of *G. nigrocaulis* (Vu et al., unpublished).

**Table 3:** **List of the top differentially expressed genes in Genlisea traps**.

**Feature ID**	**Trap EV**	**Fold change**	***P*-value**	**Description**	**EC**
**TRANSCRIPTION FACTORS AND CELL DIFFERENTIATION**					
Gnig_g5834	3.24	71.93	0.01	Bel1-like homeodomain protein 2	–
Gnig_g2911	3.99	54.57	0.02	Homeobox-leucine zipper protein hat14-like	–
Gnig_g10300	2.83	3.12	0.03	Low quality protein: uncharacterized loc101213316	–
Gnig_g6470	5.81	2.66	0.05	Ethylene-responsive transcription factor erf113	
Gnig_g13661	4.93	3.05	5.51E-4	Wrky transcription factor 22	–
Gnig_g7714	3.78	2.51	0.03	Fasciclin-like arabinogalactan protein 11-like	–
Gnig_g3900	3.59	2.13	3.72E-3	Homeobox-leucine zipper protein anthocyaninless 2-like	–
Gnig_g11132	1.32	−2.88	0.04	Mitochondrial import inner membrane translocase subunit tim-10 isoform 2	–
Gnig_g3165	0.63	−3.84	0.02	Wuschel-related homeobox 1-like	–
Gnig_g6054	1.29	−4.03	0.04	Transcription factor tcp15-like	–
Gnig_g8736	2.16	−2.11	0.05	Zf-hd homeobox protein at4g24660-like	
**DNA REPLICATION, DNA REPAIR MECHANISM, RESPONSE TO OXIDATIVE STRESS**					
Gnig_g10176	1.44	6.7	1.07E-3	Dna topoisomerase 2-like	EC:5.99.1.3
Gnig_g6886	0.34	4.2	0.03	Probable atp-dependent rna helicase ddx11-like	–
Gnig_g465	7.5	2.42	0.01	Peroxidase 4	EC:1.11.1.7
Gnig_g6251	2.81	26.98	0.02	Gag-pol polyprotein	–
Gnig_g7174	4.18	6.26	0.01	Hypothetical retrotransposon	–
Gnig_g9007	−1.24	5.84	0.04	Retrotransposon ty3-gypsy subclass	
Gnig_g12518	2.29	2.09	0.02	Dna primase small subunit-like	–
Gnig_g5726	0.92	−2.12	0.05	Cyclin-sds-like	–
**HORMONE METABOLISM**					
Gnig_g1638	4.5	7.84	0.05	Gibberellin 20-oxidase	EC:1.14.11.0
**TRANSPORT ACTIVITIES**					
Gnig_g2161	4.42	7.36	0.04	Ammonium transporter 3 member 1-like	–
Gnig_g1022	5.46	4.17	0.04	White-brown-complex abc transporter family	EC:3.6.3.28
Gnig_g12092	2.61	3.57	9.65E-4	Protein sensitive to proton rhizotoxicity 1-like	–
Gnig_g2102	3.04	2.39	0.01	Probable metal-nicotianamine transporter ysl7-like	–
Gnig_g2809	4.19	2.19	0.04	Mate efflux family protein dtx1-like	
Gnig_g2832	1.18	−2.25	0.01	Vacuolar amino acid transporter 1-like	–
Gnig_g14845	0.99	−2.3	9.78E-3	Cation h(+) antiporter 15-like	–
Gnig_g3612	0.85	−4.34	0.02	Peptide transporter ptr1	–
**HYDROLASE ACTITIVITIES**					
Gnig_g53	4.38	7.07	0.01	Pollen allergen	
Gnig_g2873	2.46	2.34	0.03	Polyphenol oxidase	–
Gnig_g11834	1.22	−4.41	0.03	Subtilisin-like protease	EC:3.4.21.0
**ENERGY METATBOLISM, MITOCHONDRIA ACTITIVITIES**					
Gnig_g71	4.02	2.59	0.02	Nadph oxidase	EC:1.6.3.0
Gnig_g2907	5.85	2.28	0.04	Duf246 domain-containing protein at1g04910-like	–
Gnig_g9092	6.2	2.02	1.00E-3	Cytochrome p450 86b1	–
Gnig_g3919	1.99	2.22	0.02	Cytochrome p450	
Gnig_g69	5.11	2.01	0.02	Nadph oxidase	EC:1.6.3.1; EC:1.11.1.7
Gnig_g13497	0.95	−4.2	0.02	Formyltetrahydrofolate deformylase	EC:3.5.1.10; EC:2.1.2.0
Gnig_g10196	0.19	−10.66	0.03	Cysteine desulfurase mitochondrial	
**PHOTOSYNTHESIS OR CHLOROPLAST ACTIVITIES**					
Gnig_g8373	−0.05	56.93	0.04	Ribulose- -bisphosphate carboxylase oxygenase large subunit	EC:4.1.1.39
Gnig_g825	2.51	−2.04	0.04	Peptidyl-prolyl cis-trans isomerase chloroplastic-like	–
Gnig_g12417	3.06	−2.4	0.01	Lipoxygenase 2	EC:1.13.11.12
Gnig_g5094	0.82	−2.4	0.02	Uracil phosphoribosyltransferase-like	EC:2.4.2.9
Gnig_g11978	0.86	−2.81	0.01	Uridine kinase -like	EC:2.7.1.48
Gnig_g1617	1.33	−3.08	0.04	Heme-binding-like protein chloroplastic-like	–
Gnig_g1973	3.07	−3.13	0.03	Carbonic chloroplastic-like isoform x1	–
Gnig_g15746	0.09	−52	0.03	Photosystem ii 47 kda protein	–
**OTHER OR UNKNOWN FUCTIONS**					
Gnig_g12094	2.42	5.65	0.05	Ring-h2 finger protein atl57-like	–
Gnig_g9230	3.98	3.36	0.02	Hypothetical protein POPTR_0011s00710g	
Gnig_g13075	−1.73	3.3	0.01	Low quality protein: udp-rhamnose:rhamnosyltransferase 1-like	–
Gnig_g5322	5.2	2.52	0.02	e3 ubiquitin-protein ligase pub23-like	
Gnig_g11156	1.27	2.33	0.05	Ubiquitin conjugating enzyme	EC:6.3.2.19
Gnig_g9986	1.23	2.21	0.04	Afadin- and alpha-actinin-binding protein a isoform x2	–
Gnig_g7098	2.4	2.12	0.03	Hypothetical protein POPTR_0001s33000g	
Gnig_g2799	2.08	−2	0.04	Conserved hypothetical protein	
Gnig_g5356	2.1	−2.14	0.04	PREDICTED: uncharacterized protein LOC100254610	
Gnig_g7417	2	−2.14	8.02E-4	Structural constituent of ribosome	
Gnig_g12123	0.9	−2.14	0.02	Hydroxycinnamoyl-coenzyme a shikimate quinate hydroxycinnamoyltransferase	–
Gnig_g5731	1.86	−2.26	0.04	Probable inactive receptor kinase at1g48480	–
Gnig_g15181	2.52	−2.34	0.03		
Gnig_g15672	1.29	−2.37	2.35E-3	Low quality protein: promoter-binding protein spl10	–
Gnig_g7001	0.96	−2.42	0.04	Histone acetyl transferase gnat myst 101	–
Gnig_g10961	1.04	−3.16	0.01	Une1-like protein	
Gnig_g5918	1.03	−3.24	0.05	Probable gpi-anchored adhesin-like protein pga55	
Gnig_g8882	1.1	−3.42	9.24E-3	Probable serine threonine-protein kinase rlckvii-like	–
Gnig_g7495	0.55	−4.1	0.04	e3 ubiquitin-protein ligase ring1-like isoform 1	
Gnig_g10879	0.3	−12.68	1.66E-3	PREDICTED: uncharacterized protein YNL011C	–
Gnig_g13974	3.32	−46.21	0.01	Tetratricopeptide repeat-like superfamily protein	
Gnig_g9460	0.02	−69.06	0.03	Kinesin-1-like	–
Gnig_g8274	0.33	−137.05	0.02	Desiccation-related protein pcc13-62-like	–

Among the 69 most differentially expressed genes, GO term annotations in either “biological process”, “molecular function” or “cellular component” could be assigned to 63 genes. Comparison of the biological processes represented by the genes with up- or down regulated expression between *G. nigrocaulis* traps and leaves indicates a switch from photosynthesis and chloroplast activities in leaves toward respiratory and mitochondrial activities in traps (Table 3). In chlorophyll-free rhizophylls, we observed a down-regulation of photosystem II protein (Gnig_g15746) and 6 other genes working in chloroplast (Gnig_g825, Gnig_g12417, Gnig_g5094, Gnig_g11978, Gnig_g1617, and Gnig_g1973). The only strongly up-regulated chloroplast gene encodes the large subunit of ribulose-biphosphate carboxylase oxygenase (Rubisco, Gnig_g8373), which participates in CO_2_ fixation in the Calvin cycle. On the other hand, two cytochrome P450 (Gnig_g9092, Gnig_g3919) and two NADH oxidases (Gnig_g71, Gnig_g69), which contribute to generate ATP via the respiratory pathway, were up-regulated. Interestingly, NADH oxidases are probably used to generate superoxide and further reactive oxygen species for prey digestion in Genlisea traps (Albert et al., 2010). Similar to other higher plants (Mittler et al., 2004), in response to oxidative stress, Genlisea trap cells display a high expression level of cytochrome P450 (Gnig_g9092, Gnig_g3919), peroxidase (Gnig_g465). Oxidative stress, as shown in *C. reinhardtii*, confers translational arrest of Rubisco (Cohen et al., 2005). This may explain the high abundance of Rubisco (Gnig_g8373) transcipts in Genlisea trap cells. Interestingly, in response to DNA damage, DDX11-like RNA helicase (Gnig_g6886), DNA topoisomerase (Gnig_g10176) and DNA primase (Gnig_g12518) together with genes required for retrotransposition (Gnig_g6251, Gnig_g7174, and Gnig_g9007) were elevated. A retrotransposition burst can be induced by different endogenous and environmental challenges including oxidative stress in plant (Mhiri et al., 1997) and other systems such as human (Giorgi et al., 2011) and yeast (Ikeda et al., 2001). Under oxidative stress, elevated DNA double strand break (DSB) repair sites at retrotransposon positions and signatures of non-homologous end joining repair (NHEJ) were uncovered in mouse (Rockwood et al., 2004). Surprisingly, the cyclin-SDS (SOLO DANCERS)-like gene (Gnig_g5726) which is involved in DSB repair via homologous recombination (De Muyt et al., 2009) was suppressed in Genlisea traps, suggesting that NHEJ is the main repair mechanism for DSBs in Genlisea trap cells.

It has been suggested that Utricularia traps serve to enhance the acquisition of P rather than of N (Sirová et al., 2003, 2009; Ibarra-Laclette et al., 2011). This was used to explain why N concentrations (both NH_4_-N and organic dissolved N) in Utricularia traps are consistently high, even in species growing in highly oligotropic waters with low prey-capture rates (Sirova et al., 2014). In *G. nigrocaulis* traps, however, we detected high up-regulation for ammonium transporter (Gnig_g2161), nitrate transporter (Gnig_g12092), amino acid transporter (Gnig_g1022), and oligopeptide transporter (Gnig_g2102) transcripts. Moreover, three transcription factors (Gnig_g5834, Gnig_g2911, and Gnig_g10300), likely involved in cellular nitrogen metabolism were the most up-regulated genes in Genlisea traps. Likely, Genlisea plants absorb N-nutrients via carnivory. For P-nutrient demand of Genlisea plants, there are four up-regulated genes, of which proteins are predicted to have acid phosphatase activity (EC:3.1.3.2/0), including Gnig_g15303, Gnig_g1090, Gnig_g9666 and Gnig_g2820. Although six (inorganic) phosphate (co)transporters (Gnig_g10119, Gnig_g1924, Gnig_g1927, Gnig_g1929, Gnig_g6455 and Gnig_g6456) were expressed in Genlisea traps, these genes do not show a significant differential expression (Table S5). We speculate that inorganic phosphates were delivered to and actively consumed in leaf cells similarly as in rhizophyll cells. In addition to four acid phosphatases, Genlisea trap cells up-regulate seven other hydrolases (EC:3.1), but only pectinesterase (Gnig_g4571) was predicted to be secreted into extracellular region. Furthermore, the gene Gnig_g53 with similarity to extracellular pollen allergen, a member of the glycoside hydrolase family, was found to be highly up-regulated. The limited number of hydrolases found to be up-regulated, suggests that in Genlisea carnivory requires additional digestive enzymes from entrapped microbes.

## Concluding remarks

Metatranscriptomic data of Genlisea traps uncovered the diverse entrapped and alive microbe community including Bacteria, protists of the SAR group (heterokont Stramenopiles, Alveolata, and Rhizaria), green algae, microbial fungi and a large range of minute metazoans. Ribosomal RNA profiling indicates a highly dynamic structure of the trap bacterial community, reflecting their ecological importance mainly as prey of the one-way food web inside Genlisea traps. The enrichment in facultatively anaerobic bacteria suggests an occasionally interrupted anoxia environment in Genlisea digestive chambers. A high amount of superoxide and other reactive oxygen species is likely generated in Genlisea traps for killing prey and stimulates different oxidative stress responses in trap cells.

The opportunistic feeding behavior, to catch and utilize various prey, provides Genlisea plants alternative N- and P- macronutrient sources from microbes. The abundance of bacteria involved in nitrogen cycling (ammonia oxidizing, nitrite reducing and nitrogen fixation) indicates their importance for the gain of N- nutrients. In addition, various transporters for different N- forms such as ammonium, nitrate, amino acids and oligopeptides together with transcription factors involved in cellular nitrogen metabolism are highly up-regulated in Genlisea rhizophylls. Except for acidic phosphatases, only a limited range of Genlisea hydrolases were found up-regulated in the traps, suggesting that Genlisea plants rely on digestive enzymatic systems from microbes. Indeed, various hydrolases were identified from entrapped metazoan microbes, Alveolata protists, green algae and amoeboid protozoa. Among them, the cilliate *T. thermophila* is a voracious bacterial predator, while green algae, such as *C. reinhardtii*, seem to stay as commensals or inquilines inside Genlisea traps. A variety of mites, nematodes, rotifers and annelids are similarly entrapped and ingest in turn protozoans until they perish and their corpses serve themselves as nutrient. Further studies using microcosm experiments with less complex microbial community may be interesting to understand contributions of each microbe to the carnivory.

## Author contributions

HC and GV conceived and designed the study. HC and GV performed the experiments and analyzed the data. HC and GV wrote the paper with contributions from IS. AP, TS, US, and IS contributed reagents/ materials/ analysis tools. All authors read and approved the final manuscript.

### Conflict of interest statement

The authors declare that the research was conducted in the absence of any commercial or financial relationships that could be construed as a potential conflict of interest.
