# Supplementary material for: Metatranscriptome analysis reveals host-microbiome interactions in traps of carnivorous Genlisea species
Source: Front Microbiol. 2015 Jul 14;6:526. doi: 10.3389/fmicb.2015.00526 (PMC4500957; doi:10.3389/fmicb.2015.00526)
Supplement: Supplementary file 6 [file Image1.PDF]

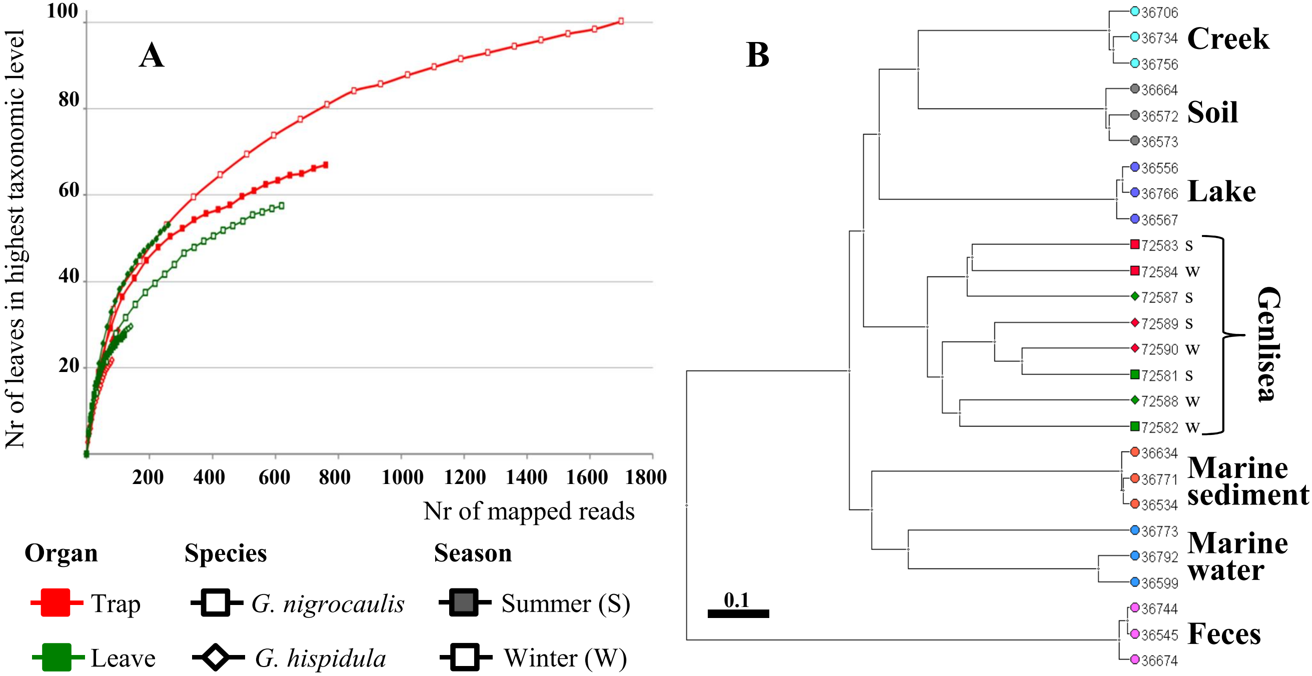

Figure S1: Diversity and dissimilarity of the bacterial communities in Genlisea samples. (A) Taxonomy rarefaction curves for each Genlisea sample with information of all 403 bacterial taxa. (B) The UPMA cluster dendrogram was generated using a Bray-Curtis dissimilarity matrix applied to abundance of all 624 bacterial taxa identified in Genlisea and reference environmental samples. Sample information can be found in Table 1 and Table S1.
