# Supplementary material for: Metatranscriptome analysis reveals host-microbiome interactions in traps of carnivorous Genlisea species
Source: Front Microbiol. 2015 Jul 14;6:526. doi: 10.3389/fmicb.2015.00526 (PMC4500957; doi:10.3389/fmicb.2015.00526)
Supplement: Supplementary file 7 [file Image2.PDF]

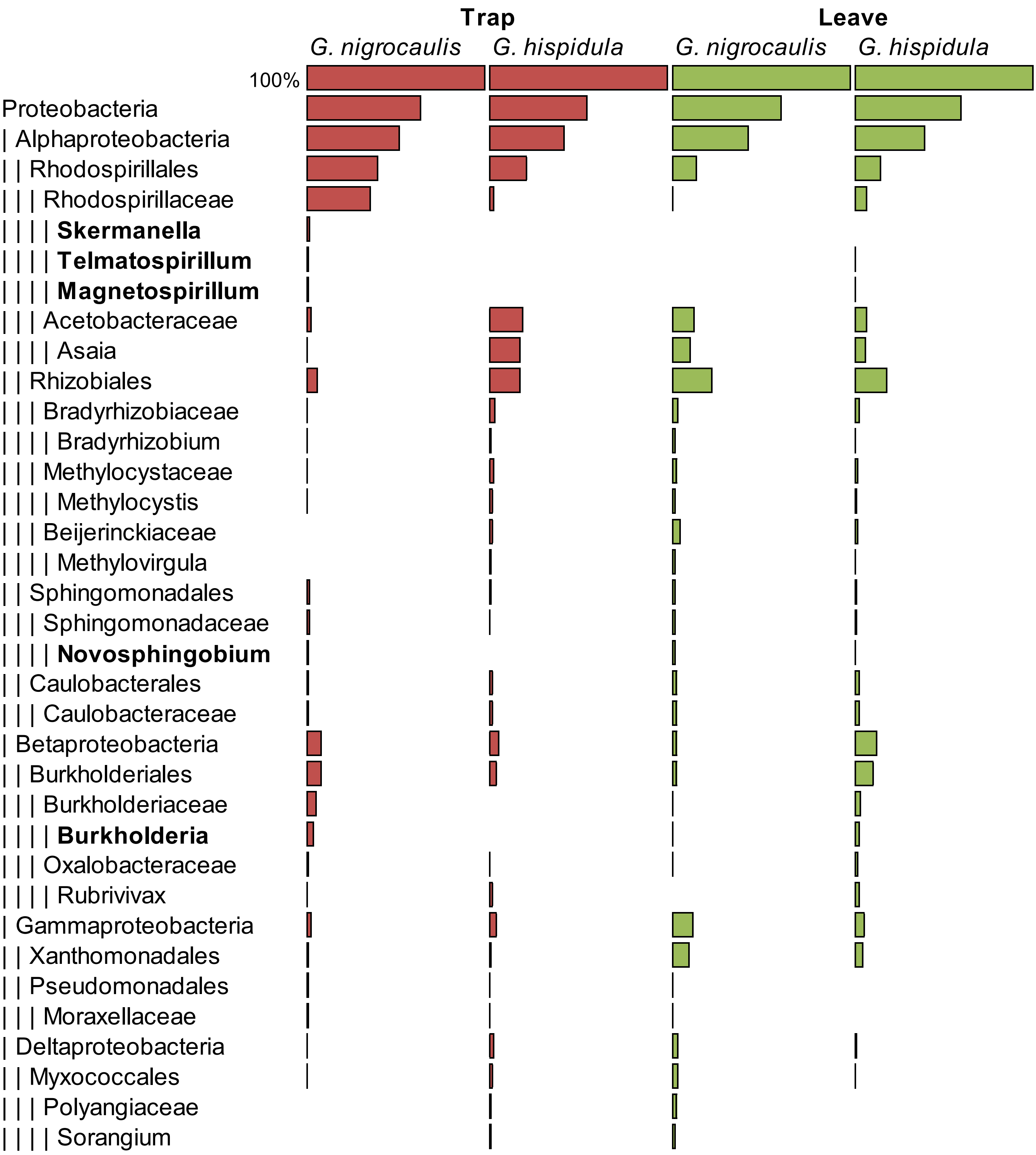
Trap
Leave
G. nigrocaulis
G. hispidula
G. nigrocaulis
G. hispidula
100%
Proteobacteria
| Alphaproteobacteria
| | Rhodospirillales
| | | Rhodospirillaceae
| | | | Skermanella
| | | | Telmatospirillum
| | | | Magnetospirillum
| | | Acetobacteraceae
| | | | Asaia
| | Rhizobiales
| | | Bradyrhizobiaceae
| | | | Bradyrhizobium
| | | Methylocystaceae
| | | | Methylocystis
| | | Beijerinckiaceae
| | | | Methylovirgula
| | Sphingomonadales
| | | Sphingomonadaceae
| | | | Novosphingobium
| | Caulobacterales
| | | Caulobacteraceae
| Betaproteobacteria
| | Burkholderiales
| | | Burkholderiaceae
| | | | Burkholderia
| | | Oxalobacteraceae
| | | | Rubrivivax
| Gammaproteobacteria
| | Xanthomonadales
| | Pseudomonadales
| | | Moraxellaceae
| Deltaproteobacteria
| | Myxococcales
| | | Polyangiaceae
| | | | Sorangium

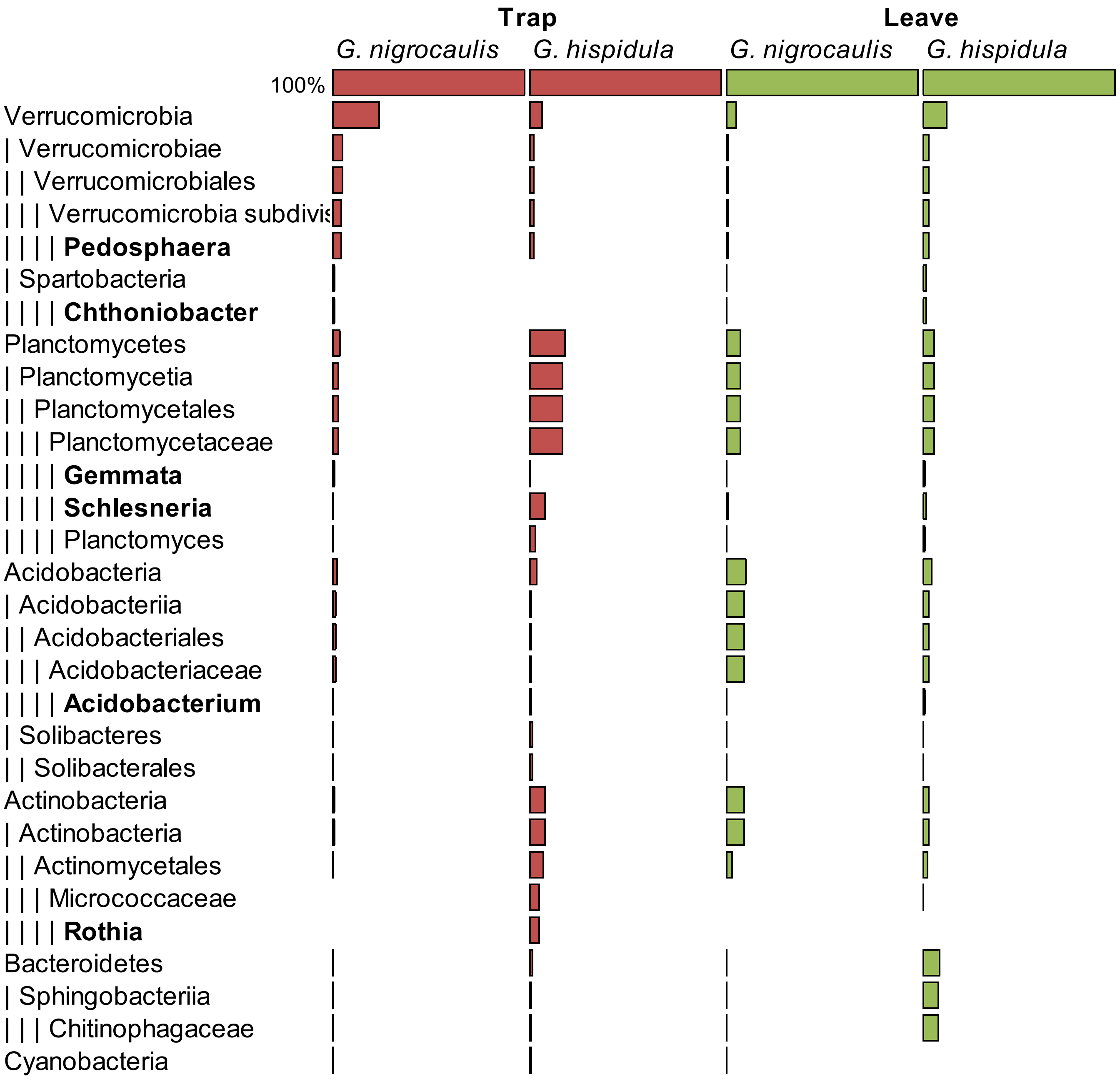

Figure S2: Relative abundance (percentage of mapped reads) of abundant bacterial taxa (>1%) in Genlisea traps and corresponding values in Genlisea leave samples. Bacterial genera that appear in the active-microbiome of Genlisea traps are in bold.
